# Changes in maternal feelings for children with autism spectrum disorder after childbirth: The impact of knowledge about the disorder

**DOI:** 10.1371/journal.pone.0201862

**Published:** 2018-08-02

**Authors:** Sarah Tomiyama, Mitsuru Kikuchi, Yuko Yoshimura, Chiaki Hasegawa, Takashi Ikeda, Daisuke N. Saito, Hirokazu Kumazaki, Nobushige Naito, Yoshio Minabe

**Affiliations:** 1 Research Center for Child Mental Development, Kanazawa University, Kanazawa, Japan; 2 Division of Socio-Cognitive-Neuroscience, Department of Child Development United Graduate School of Child Development, Osaka University, Kanazawa University, Hamamatsu University School of Medicine, Chiba University and University of Fukui, Kanazawa, Japan; 3 Department of Psychiatry and Neurobiology, Graduate School of Medical Science, Kanazawa University, Kanazawa, Japan; 4 Institute of Human and Social Sciences, Kanazawa University, Kanazawa, Japan; Chiba Daigaku, JAPAN

## Abstract

The social interactions between caregivers and their children play a crucial role in childhood development; therefore, caregivers’ feelings for children are critical for the development of social minds. Mothers of children with autism spectrum disorder (ASD) are known to experience higher levels of stress. However, knowledge regarding mothers’ feelings for their children before receiving a clinical diagnosis is limited. This study retrospectively investigated the time course of mothers’ feelings from the time of birth and the effect of protective factors. The participants were 5- to 8-year-old children with an ASD diagnosis and their mothers. The mothers of the children with ASD had less positive feelings toward their children than the mothers of the typically developed (TD) children before receiving a clinical diagnosis. Intriguingly, prior knowledge of ASD may relieve maternal mental distress during the child-rearing years and at the time of diagnosis.

## Introduction

Mothers’ emotions impact effective parenting during infancy. Strong negative emotions in parents may disrupt cognition and decision making, cause excessive interference with their child, and induce tendencies to take actions that overwhelm processes that normally control negative emotions; additionally, strong negative emotions lead to negative expressive behaviors, and vice versa [1]. The social interaction between mothers and their children plays an important role in development during infancy; consequently, a mother's feelings for her children are extremely important to the development of the child’s social mind. The emotional interactions between a mother and her child are induced by their behaviors, and such interactions play a crucial role in forming social minds. Intriguingly, recent neuroimaging studies demonstrated the neural responses which underlie this process [2–4]. Negative emotions of parents are known to cause child development problems [1, 5]. If parents’ negative emotions toward their children are prominent, their children's social capacity tends to be low, and the risk of behavioral problems and other developmental disorders tends to be high [1, 5]. More recent research suggests that negative expression that reflects mothers’ affective sensitivity to aversive child behavior promotes interaction patterns in children that place children at risk for adjustment problems [6]. Therefore, it is important to relieve the negative feelings induced by children, especially for caregivers of children with developmental disorders who tend to have difficult behaviors.

Autism spectrum disorder (ASD) is a neurodevelopmental disorder characterized by impairment in social interaction, impairment in communication, restricted interests, and repetitive behavior. Parents of children with ASD are more likely to have a depressive mood and experience stress-related physical distress than parents who care for typically developed (TD) children [7]. According to a recent longitudinal study, the psychological condition of the parents is associated with the social ability of their children with ASD [8]. Notably, emotional acceptance of ASD could be a protective factor against parenting stress and a depressive mood [9]. Less severe ASD symptoms are expected to enhance the ease of embracing acceptance of the diagnosis among caregivers. However, a recent study revealed the opposite results [9]. The subtler the symptoms, the less attention the parents are likely to pay to the disorder. Therefore, we should pay more attention to the acceptance of ASD children without intellectual disability by their parents. Regarding children with ASD without intellectual disability, a recent study demonstrated that parents of children with high-functioning ASD exhibit higher levels of parenting stress and a lower quality of life compared to parents of TD children [10].

On the other hand, some parents show psychological resilience (i.e., diminished psychological distress) after their children receive a diagnosis of a chronic medical condition [11]. These parents overcome the initial impact of receiving the diagnosis and achieve an attitude of acceptance [12]. Parents who had resolved their emotions regarding the diagnosis of a chronic medical condition reported less stress [12] and received more social support [13] than parents who had not. Therefore, the emotional acceptance of the diagnosis must be a protective factor against distress in caregivers of a child with ASD.

The positive effect of early intervention for children with ASD has been demonstrated [14–18]. In addition to developments in early intervention, early diagnostic tools have been developed [19, 20]. When their children are diagnosed, some parents experience increased levels of self-blame and despair about the diagnosis, and their mental health worsens [9]; therefore, subsequent interventions should fully consider the mothers’ emotions regarding the diagnosis of ASD [21].

However, in the case of ASD without intellectual disability, studies examining how mothers feel about their children when they notice abnormalities in their children and/or receive the clinical diagnosis of their children are limited. Moreover, studies investigating how the mothers’ feelings for their children change before receiving a clinical diagnosis are lacking. The first purpose of this study is to investigate the time course of mothers’ feelings for their children, including the period before they received a clinical diagnosis. Accordingly, we retrospectively compared the time course of mothers’ feelings (i.e., 0, 18 and 36 months of the child’s chronological age in addition to the time of the survey) between mothers of TD and ASD children without intellectual disability. We hypothesized that the mothers of children with ASD had less positive feelings toward their children than the mothers of TD children during the child-rearing years. The second purpose of this study is to investigate the effect of 3 possible protective factors (i.e., confidence in knowledge about ASD, family resources and social support) on the time course of the mothers’ feelings for their children. For the latter analysis, we employed time points related to the diagnostic event (i.e., zero months of age, the time abnormalities were recognized in their children, the time the children received the diagnosis of ASD, and the time of the survey). We hypothesized that these three protective factors relieve maternal mental distress during the child-rearing years.

## Materials and methods

### Participants

All participants were recruited from public nursery schools in Kanazawa City and Kanazawa University’s Hospital. Initially, 73 children and their mothers chose to voluntarily participate in this study. The clinically recruited children were diagnosed by a clinical psychiatrist and a clinical psychologist with more than 5 years of clinical experience in the diagnosis of ASD using the Autism Diagnostic Observational Schedule–Generic (ADOS) [22], the Diagnostic Interview for Social and Communication Disorders (DISCO) [23], and the DSM-IV [24] criteria at the time of participation in this study. ASD children were included in this study if they met the diagnosis criteria for childhood autism, atypical autism or Asperger’s syndrome with DISCO, and/or the ADOS criteria for the autism spectrum. The exclusion criteria for the children included known hearing loss or a central nervous system involvement other than autism, a low birth weight (< 1500 g) (n = 3) and intellectual disability < 70 based on the mental processing scale of the Kaufman Assessment Battery for Children (K-ABC) [25] (n = 8). The final clinical group consisted of 30 children with ASD (25 males, 5 females) aged 63–111 months and their mothers (Table 1). The control group included 32 TD children (19 males, 13 females) aged 61–79 months and their mothers (Table 1). All TD children and their parents had no prior or current developmental, learning, or behavioral problems as reported on a questionnaire completed by the parents. The parents agreed to their child’s participation in the study with full knowledge of the experimental nature of the research study. Written informed consent was obtained prior to participation. The Ethics Committee of Kanazawa University Hospital specifically approved this study, and all procedures were performed in accordance with the Declaration of Helsinki.

**Table 1:** Demographic characteristics of all participants.

	TD	ASD	*t*	*p*
***Mothers***				
Number of subjects	32	30		
Years of age	38.1 (31–45)	38.1 (31–50)	0.0	n.s.
JART (Estimated IQ)	100.6 (7.8)	103.4 (8.6)	-1.3	n.s.
SDS	35.9 (5.2)	41.4 (9.0)	-2.9	.006
STAI trait	39.8 (8.7)	45.9 (11.9)	-2.3	.024
QOL (Average)	3.7 (0.5)	3.4 (0.6)	2.2	.029
QOL subscore I (Physical)	3.9 (0.6)	3.4 (0.7)	2.4	.019
QOL subscore II (Psychological)	3.5 (0.5)	3.3 (0.8)	1.5	n.s.
QOL subscore III (Social)	3.8 (0.5)	3.4 (0.6)	2.8	.006
QOL subscore IV (Environment)	3.6 (0.6)	3.4 (0.6)	1.3	n.s.
QOL subscore V (General)	3.6 (0.7)	3.2 (0.8)	2.3	.026
SES	42.2 (8.4)	42.7 (6.0)	-0.3	n.s.
History of psychiatric treatment (yes/no)	3/29	13/17	9.3*	0.002
***Children***				
Gender (male/female)	19/13	25/5		
Chronological age (months)	70.2 (61–79)	79.8 (63–111)	-3.5	.001
Birth weight (g)	3038.2 (312.9)	3100.1 (237.9)	-0.9	n.s.
SRS total *T*-score	47.6 (7.2)	70.4 (11.5)	-9.4	P < .001
ADHD-RS	5.1 (5.0)	21.5 (10.9)**	-7.0	P < .001
SDQ	8.8 (4.5)	18.5 (5.6)**	-7.3	P < .001
K-ABC Mental Processing Scale	105.7 (13.7)	96.8 (15.0)	2.4	.018
PVT-R	10.5 (3.1)	9.3 (3.6)	1.4	n.s.

JART, the Japanese version of the National Adult Reading Test. SDS, the Zung Self-Rating Depression Scale. STAI, the State-Trait Anxiety Inventory. QOL, World Health Organization Quality of Life. SES, Socioeconomic status using the Hollingshead Index. SRS, Social Responsiveness Scale. ADHD-RS, The ADHD Rating Scale. SDQ, The strengths and difficulties questionnaire. K-ABC, Kaufman Assessment Battery for Children. PVT-R, the Picture Vocabulary Test-Revised. The values represent the mean (range or standard deviation) of each variable. n.s., not significant.

*, *X*^2^ value.

**, n = 25.

### Psychological tasks and questionnaire completed by the mothers

**Fig 1:**
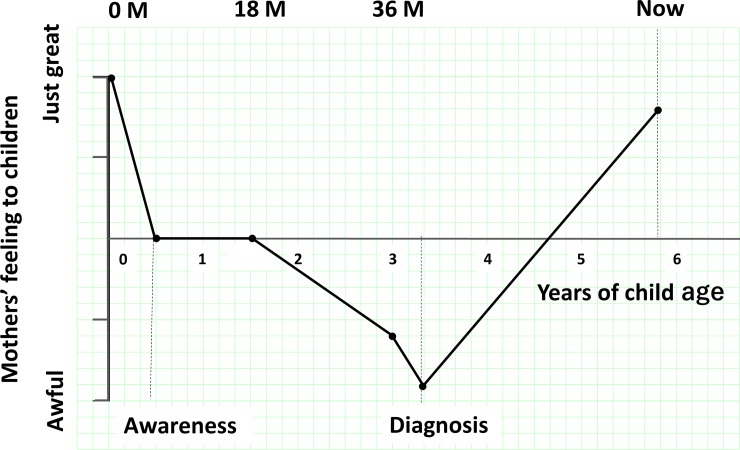
An example of the VAS scores of mothers’ feelings for their ASD children drawn on graph paper.

We conducted a semistructured interview with the mothers participating in the survey by one of the author who had a clinical experience for young children more than 8 years. We asked them to rate their feelings for their children from negative to positive using a visual analog scale (VAS; from -10 to 10). (Fig 1) The average interview time was 37 minutes. In addition, retrospectively, we asked the mothers about their feelings when their child was 0, 18 and 36 months of age and at the time of the survey. We asked the mothers of the children with ASD about their feelings for their children during an event (i.e., when they noticed an abnormality in their children and/or when they received the diagnosis of ASD). We also asked the mothers about possible factors protective against caregivers’ psychological distress associated with the diagnosis of ASD in their children (i.e., knowledge about ASD, family resources and social support). Regarding factor 1 (mothers’ confidence in knowledge about ASD), the mothers rated their degree of confidence in their knowledge about ASD before their child was diagnosed using a five-point rating scale (i.e., -2 = they did not know about ASD at all, -1 = they did not know much, 0 = neither, 1 = they have slight knowledge, 2 = they were very knowledgeable about ASD). Regarding factor 2 (family resources), we divided the types of family members who had supported the mothers into three categories (husbands, children’s grandparents and mothers’ siblings). We grouped the mothers according to the number of categories. Eventually, the number of groups was two (one category or two categories). Regarding factor 3 (social support), we divided the mothers into 3 groups based on when the mothers started to regularly visit institutions specialized in child development (i.e., 1, they started receiving social support before the clinical diagnosis; 2, they started receiving social support after the clinical diagnosis; and 3, they have not received social support).

In all mothers, we also assessed the level of anxiety, depressive symptoms and quality of life (QOL) using the State-Trait Anxiety Inventory (STAI) [26], the Zung Self-rating Depression Scale (SDS) [27], and the World Health Organization Quality of Life (WHOQOL26) [28]. The Full IQ score (FSIQ) was estimated using the Japanese version of the National Adult Reading Test (JART) [29]. The socioeconomic status was estimated using the Hollingshead Index (SES) [30].

### Psychological tasks and questionnaire regarding the children

The autistic traits of the children were quantitatively assessed by the parents using the Japanese version [31] of the social responsiveness scale (SRS) [31, 32]. Higher scores on the SRS indicate a higher degree of social impairment. The raw scores of the SRS were converted to *T*-scores (with a mean of 50 and a standard deviation of 10) for gender. The ADHD (attention deficit hyperactivity disorder) Rating Scale (ADHD-RS) [33] was employed to assess the comorbid symptoms of ADHD. Receptive vocabulary and comprehension were measured in each child using the Picture Vocabulary Test-Revised (PVT-R) [34]. K-ABC [25] was employed to estimate the intelligence level of the children. To assess the psychological adjustment, the strengths and difficulties questionnaire (SDQ) [35] was employed.

### Statistical analysis

#### Comparison of mothers’ background at the time of survey

First, to investigate the mothers' background, which may affect their emotions toward their children, we compared the social and mental conditions (STAI trait, SDS, WHOQOL26, FSIQ, SES, and age) of the mothers of the children with ASD and the mothers of the TD children at the time of the survey using an unpaired t-test (two-tailed). The significance level was set at *P* < 0.05 for all statistical analyses.

#### Comparison of mothers’ feelings for their children between the mothers of the TD and ASD children

Second, to investigate the time course of the mothers’ feelings for their children before they received a clinical diagnosis, we compared the time course of the mothers’ feelings (0, 18 and 36 months of age in addition to the time of the survey) between the mothers of the children with ASD and those of the TD children. We employed a two-way ANOVA in which “diagnosis; 2 levels (1, TD and 2, ASD)” was the between-group factor and “time; 4 levels (1, zero month of age; 2, eighteen months of age; 3, thirty-six months of age; and 4, the time of the survey)” was the within-group factor. In this analysis, we excluded two participants who received a clinical diagnosis before thirty-six months of age as we focused on the mothers’ feelings before the diagnosis. Therefore, the clinical group consisted of 28 children with ASD and their mothers (S1 Table). The control group included 32 TD children and their mothers (Table 1). If a significant main group effect or interaction was observed in the post hoc analysis, we employed an unpaired t-test between the groups at each time point. The significance level was set at *P* < 0.05 for all statistical analyses.

In addition, to consider the possible confounding factors, we added a two-way ANCOVA in which “diagnosis; 2 levels (1, TD and 2, ASD)” was the between-group factor, “time; 4 levels (1, zero months of age; 2, eighteen months of age; 3, thirty-six months of age; and 4, the time of the survey)” was the within-group factor, and intellectual level (K-ABC Mental Processing Scale), gender (male = 0, female = 1) in children, depressive mood (SDS score), anxious trait (STAI-trait score), and history of psychiatric treatment (without history = 0, with history = 1) in the mothers served as the covariates.

#### Effect of the three possible factors on the mothers’ feelings for their children with ASD

Third, to investigate the protective effect of the three possible factors on the mothers’ psychological distress associated with the diagnosis of ASD, we employed a two-way ANOVA in which “time; 4 levels (1, zero months of ages; 2, when they noticed abnormalities in their children; 3, when they received the diagnosis of ASD; and 4, the time of the survey)” was the within-group factor and “each possible protective factor” (confidence in their knowledge of ASD, family resources and social support) was the between-group factor. The levels of protective factor 1 (confidence in knowledge of ASD) were 1 (they did not know at all), 2 (they did not know much), 3 (they knew a little), and 4 (they were very knowledgeable). The levels of factor 2 (family resources; the number of categories that can support the participant) were 2 (1, husbands or grandparents of the child and 2, husbands and grandparents of the child or siblings of the mother). The levels of factor 3 (social support) were 1 (they started receiving social support before the clinical diagnosis), 2 (they started receiving social support after the clinical diagnosis), and 3 (they have not received social support). In this analysis, we included all participants as shown in Table 1. If a significant main group effect or interaction was observed in the post hoc analysis, we employed an unpaired t-test or one-way ANOVA among the groups at each time point. The significance level was set at *P* < 0.05 for all statistical analyses.

#### Additional background effects on mothers’ feelings for their children at the time of diagnosis

Fourth, to confirm the possible effect of the mothers’ (i.e., FSIQ, age, and degree of knowledge about ASD) and children’s (i.e., SRS, SDQ, ADHD-RS, PVT-R, K-ABC mental processing scale, birth weight, months of age when they displayed abnormalities, and months of age when they received the diagnosis of ASD) background, Spearman's rank correlation coefficient was employed to investigate the relationships between the mothers’ feelings for their children at the time of diagnosis and the above possible background factors. The significance level was set at *P* < 0.05 for all statistical analyses.

## Results

### Comparison of maternal mental conditions between the mothers of the TD and ASD children at the time of the survey

As shown in Table 1 and S1 Table, the unpaired t-test (two-tailed) revealed a significant difference in the STAI-trait score, SDS score, WHOQOL26 several subscores and history of psychiatric treatment (see statistical values in Table 1 and S1 Table), but no significant difference was observed in FSIQ, SES, and age between the mothers of the children with ASD and those of the mothers of the TD children (see statistical values in Table 1 and S1 Table).

### Comparison of mothers’ feelings for their children between the mothers of the TD and ASD children

**Fig 2:**
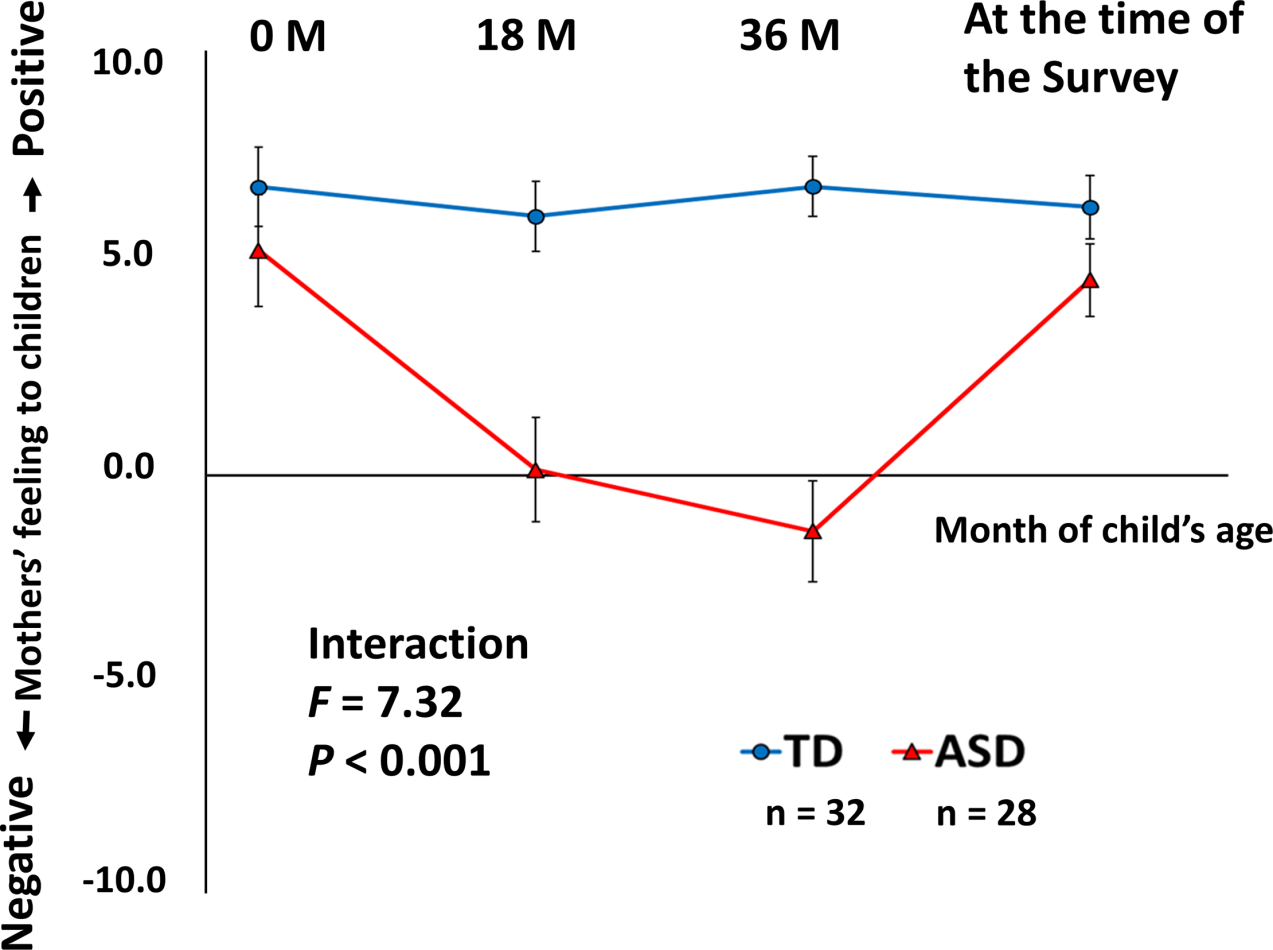
VAS scores of mothers’ feelings for their children. Red triangles indicate parents who have ASD children (*n* = 28). Blue circles indicate parents who have TD children (*n* = 32). A two-way ANOVA revealed a significant interaction between diagnosis and time. The values indicate the mean. Error bars represent the standard errors.

The two-way ANOVA revealed a significant interaction between diagnosis and time (*df* = 3, 174, *F* = 7.322, *P* < 0.001). As shown in Fig 2, the mothers of the children with ASD had less positive feelings for their children than the mothers of the TD children during the toddler period (18 to 36 months of age, i.e., the time before the diagnosis). In the additional analysis using the two-way ANCOVA, which considered the possible confounding factors, this significant interaction between diagnosis and time remained (*df* = 3, 159, *F* = 8.625, *P* < 0.001). The post hoc unpaired t-test revealed significant differences between the groups at eighteen months of age (*df* = 58, *t* = 4.03, *P* < 0.001) and thirty-six months of age (*df* = 58, *t* = 6.01, *P* < 0.001); however, no significant differences were observed at zero months of age (*df* = 58, *t* = 1.13, *P* = 0.26) and the time of the survey (*df* = 58, *t* = 1.69, *P* = 0.95).

### Effect of ASD knowledge on the mothers’ feelings for their children

**Fig 3:**
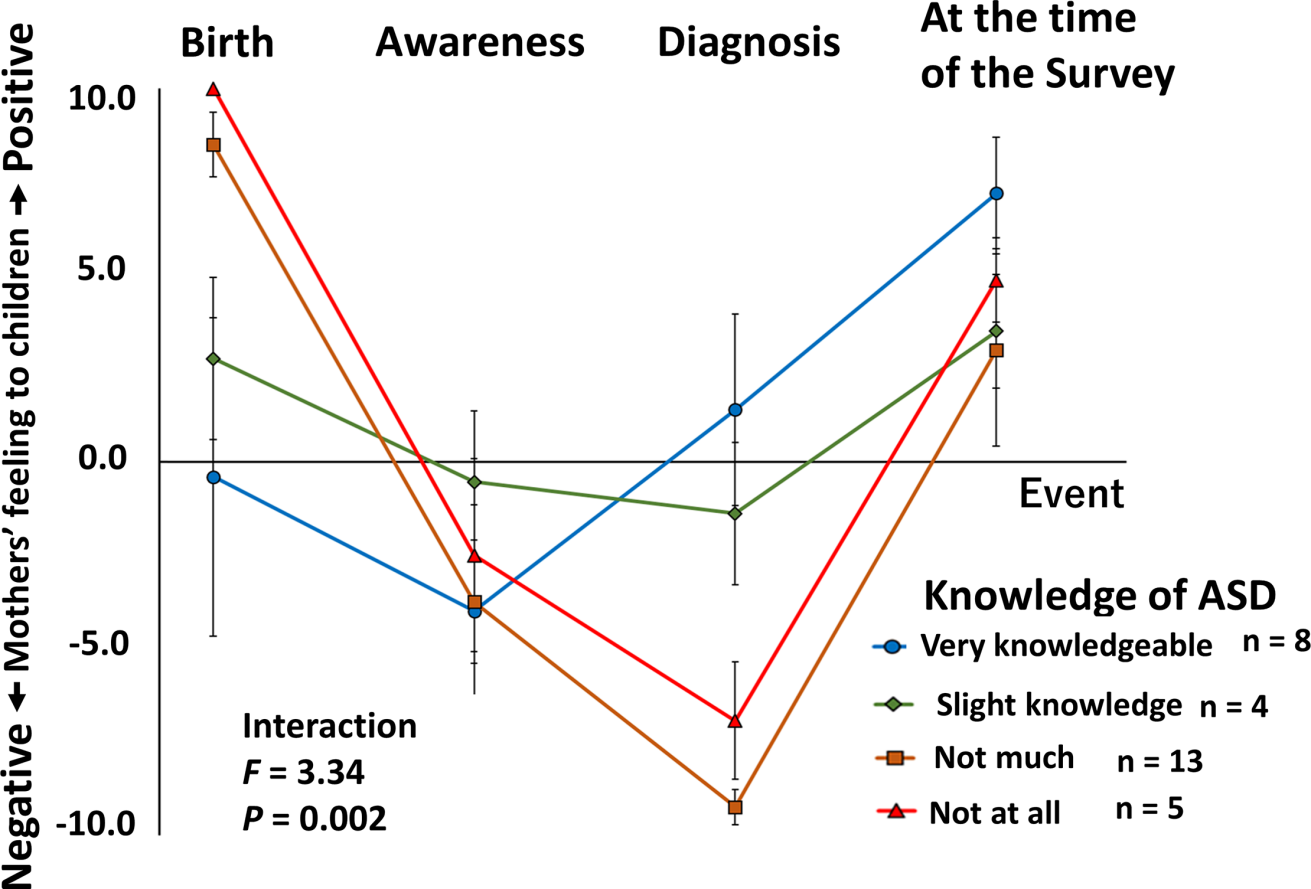
VAS scores of mothers’ feelings for their children with ASD. Group factors are the 4 levels of the mothers’ confidence in their ASD knowledge. Red triangles indicate the group in which the mothers did not know of ASD at all (*n* = 8). Brown squares indicate the group in which the mothers did not know much (*n* = 4). Green diamonds indicate the group in which the mothers have slight knowledge of ASD (*n* = 13). Blue circles indicate the group in which the mothers were very knowledgeable (*n* = 5). Two-way ANOVAs revealed significant interactions between knowledge and time (group and time points related to the diagnostic event). The values indicate the mean. Error bars represent the standard errors.

A two-way ANOVA in which “confidence in knowledge of ASD (1, they did not know at all; 2, they did not know much; 3, they knew a little; and 4, they were very knowledgeable)” was the between-groups factor and “time (1, zero months of age; 2, when they noticed abnormalities in their children; 3, when they received the ASD diagnosis; and 4, the time of the survey)” was the within-group factor was performed. For the questionnaire item “confidence in knowledge of ASD”, none of the mothers answered “neither”; therefore, this factor was given a designation of level 4. As shown in Fig 3, a significant interaction was observed between knowledge and time (*df* = 9, 78, *F* = 3.34, *P* = 0.002). Therefore, we performed a post hoc analysis to investigate the effect of confidence in ASD knowledge on the mothers’ feelings for their children at each time point and employed a one-way ANOVA in which “confidence in knowledge of ASD” was the between-group factor. A significant main effect (confidence level) was observed at two time points, i.e., zero months of age (*df* = 3, *F* = 3.59, *P* = 0.027) and the time when they received the diagnosis of ASD (*df* = 3, *F* = 4.18, *P* = 0.015). However, no significant main effect was observed at the other two following time points: the time when they noticed abnormalities in their children (*df* = 3, *F* = 0.47, *P* = 0.704) and the time of the survey (*df* = 3, *F* = 0.92, *P* = 0.444).

### Effect of family resources on the mothers’ feelings for their children

We performed a two-way ANOVA in which “family resources (1, husbands or grandparents of the children and 2, husbands and grandparents of the children or siblings of the mothers)” was the between-group factor and “time (1, zero months of age; 2, when they noticed abnormalities in their children; 3, when they received the diagnosis of ASD; and 4, the time of the survey)” was the within-group factor. No significant main effect (group; *df* = 1,28, *F* = 2.69, *P* = 0.11) or interaction between family resources and time (*df* = 3, 84, *F* = 0.37, *P* = 0.77) was observed.

### Effect of social support on the mothers’ feelings for their children

We performed a two-way ANOVA in which “social support (1, they started receiving social support before the clinical diagnosis; 2, they started receiving social support after the clinical diagnosis; and 3, they have not received social support)” was the between-group factor and “time (1, zero months of age; 2, when they noticed abnormalities in their children; 3, when they received the diagnosis of ASD; and 4, the time of the survey)” was the within-group factor. No significant main effect (group; *df* = 2,27, *F* = 0.98, *P* = 0.39) or interaction between social support and time (*df* = 6, 81, *F* = 0.19, *P* = 0.98) was observed.

### Additional background effects on mothers’ feelings for their children at the time of diagnosis

The Spearman’s rank correlation coefficients revealed a significant correlation between the VAS score (i.e., mothers’ feelings for their children) at the time of diagnosis and the confidence in their knowledge of ASD at the time of diagnosis (*ρ* = 0.54, *P* = 0.002). However, no significant correlations were observed between the VAS score and the following additional possible factors: FSIQ (ρ = 0.19, P = 0.31), age of mothers (*ρ* = -0.07, *P* = 0.73), SRS (*ρ* = -0.16, *P* = 0.41), SDQ (*ρ* = -0.18, *P* = 0.39), ADHD-RS (*ρ* = -0.01, *P* = 0.95), PVT-R (*ρ* = 0.13, *P* = 0.48), K-ABC mental processing scale (*ρ* = 0.29, *P* = 0.12), birth weight (*ρ* = -0.05, *P* = 0.37), months of age when they noticed abnormalities in their children (*ρ* = -0.04, *P* = 0.85), and months of age when they received the diagnosis of ASD (*ρ* = 0.06, *P* = 0.74).

## Discussion

The main purpose of this study was to retrospectively investigate the time course of mothers’ feelings for their children from the time of birth and the effect of possible protective factors (confidence in knowledge of ASD, family resources and social support) at various time points. During the period (18 to 36 months of age) before they received the clinical diagnosis, the mothers of the children with ASD had less positive feelings for their children than the mothers of the TD children. Regarding the 3 plausible protective factors, confidence in ASD knowledge seemed to be crucial to the mothers. Mothers who were more confident in their knowledge of ASD had less negative feelings at the time of diagnosis. In summary, the mothers of the children with ASD had less positive feelings for their children than the mothers of the TD children before they received a clinical diagnosis. Therefore, prior knowledge of the developmental disorder may relieve maternal mental distress during the child-rearing years and at the time of diagnosis.

In the present study, at the time of the survey, no difference was observed in the mothers' feelings for their children between the mothers of the TD children and those of the ASD children. The mothers who voluntarily participated in the present study were willing to deepen their understanding of ASD. Because of this background, most mothers had likely already accepted their child’s developmental disorder. Thus, the subjects in this study are considered to have selection bias toward positive feelings for their children.

Although a selection bias toward positive feelings may exist, it was difficult for the mothers to feel positive toward their children at the prediagnosis time of 18 and 36 months. Furthermore, at the time of the survey, no significant difference in the mothers' feelings for their children was observed between the mothers of the TD children and those of the ASD children; however, the QOL at the time of the survey was lower, depressive and anxious tendencies at the time of the survey were higher, and a history of psychiatric treatment was more prevalent in the mothers of the children with ASD. This finding is consistent with previous reports that the caregivers of ASD children are under stress and have a high depressive tendency [7] and a lower QOL [36], which could be improved by social supports [37]. If selection bias did not exist, the negative emotions might have been stronger, and the psychiatric symptoms may have been greater. Our results suggest that even in cases of high-functioning autism, earlier detection and support for the parents and children should be considered.

Although limited research studies have examined caregiver knowledge of ASD and its relation to caregiver stress, a recent study demonstrated that more integrated knowledge enables the parents' acceptance of the disease [38]. These results are consistent with the results of the present study. If mothers have sufficient knowledge about social development beginning in early childhood, it may be possible to prevent the adverse effects on the parent-child relationship at the time of diagnosis of ASD. Conversely, if a mother's confidence in her knowledge of ASD is insufficient, we should consider the parent-child relationship. Given the high prevalence rate of ASD (2.6%) [39], all parents should learn about ASD symptoms during young childhood for public mental health reasons.

Family and social resources have been reported to be directly associated with caregivers’ well-being [40–42]. In addition, interventions involving social support contribute to improvements in parenting stress and reductions in depression and anxiety among caregivers [43]. In the present study, however, we could not confirm this effect. This issue may be due to methodological differences and/or weak statistical power because of the small sample sizes.

According to a previous study, the anxiety of caregivers who have children with ASD is significantly higher if their knowledge and beliefs about ASD etiology are inconsistent [44]. As a historical background of ASD etiology, in the 1960s, mothers of children with ASD were called "Refrigerator mothers", and there were misunderstandings and stigmatization in the wider community that the cause of ASD was the mothers' parenting attitude. As the importance of biological genetic research has increased and the etiology of ASD is assumed to be biogenetic, the skeptical theory of the relationship between autism and parenting attitudes of the mothers has diminished. Therefore, medical knowledge of ASD must have crucial role to prevent caregivers' despair caused by stigmatization and lead to better parent-child relationships.

The present study has some limitations. First, the mothers were motivated to participate in a study that focused on ASD; thus, selection bias existed in this research. Further research studies involving birth cohorts are necessary to draw a definitive conclusion based on unbiased data. Second, it was necessary for the mothers to remember their feelings for their children retrospectively; therefore, recall bias existed in this research study. Third, we could not consider the mothers’ anxiety and depressive mood in the past period, which could be confounding factors for our results. Fourth, the present study is a novel investigation of the time course of mothers’ feelings. Therefore, without the effect size predicted by prior studies, we explore our hypothesis in a small number of subjects as a pilot study. Fifth, VAS have been thought to be a useful and simple direct method for measuring patients’ subjective pain. However, the use of a VAS to measure mothers’ feelings for their children requires validation against a detailed questionnaire. Further prospective research studies with larger sample sizes and the use of detailed questionnaires are necessary to control various confounding factors. Sixth, we did not evaluate the degree of correct knowledge the mothers had. Further studies using qualitative and quantitative evaluations of knowledge are necessary.

## Supporting information

S1 File Data sheet statistically analyzed in this study. TD, typically developed. ASD, Autism spectrum disorder. C_Gender, child gender (0 is male and 1 is female). SRS_T, total score of Social Responsiveness Scale. SDQ_T, total score of the strengths and difficulties questionnaire. Dx_Month, the month of child’s age when they received the diagnosis of ASD. Mo_age, mother’s age. Mo_QOL_Av, average of mother’s World Health Organization Quality of Life. Mo_QOL_1~5, subscore of QOL (Physical, Psychological, Social, Environment and General). Mo_Psych_history, History of psychiatric treatment (0 is no and 1 is yes).
(XLSX)

S1 Table Demographic characteristics of the participants in which two subjects are excluded for further analysis. (DOCX)

## References

[1] Dix T. The affective organization of parenting: adaptive and maladaptive processes. Psychol Bull. 1991;110:3–25. 1891517 10.1037/0033-2909.110.1.3

[2] Carver LJ, Vaccaro BG. 12-month-old infants allocate increased neural resources to stimuli associated with negative adult emotion. Developmental psychology. 2007;43:54–69. 10.1037/0012-1649.43.1.54 17201508 PMC3593093

[3] Musser ED, Kaiser-Laurent H, Ablow JC. The neural correlates of maternal sensitivity: an fMRI study. Dev Cogn Neurosci. 2012;2:428–436. 10.1016/j.dcn.2012.04.003 22652538 PMC7005764

[4] Hasegawa C, Ikeda T, Yoshimura Y, Hiraishi H, Takahashi T, Furutani N, et al Mu rhythm suppression reflects mother-child face-to-face interactions: a pilot study with simultaneous MEG recording. Sci Rep. 2016;6:34977 10.1038/srep34977 27721481 PMC5056356

[5] Eisenberg N, Gershoff ET, Fabes RA, Shepard SA, Cumberland AJ, Losoya SH, et al Mothers' emotional expressivity and children's behavior problems and social competence: mediation through children's regulation. Dev Psychol. 2001;37:475–490. 11444484 10.1037//0012-1649.37.4.475

[6] Moed A, Dix T, Anderson ER, Greene SM. Expressing negative emotions to children: Mothers' aversion sensitivity and children's adjustment. J Fam Psychol. 2017;31:224–233. 10.1037/fam0000239 27748616

[7] De Andres-Garcia S, Moya-Albiol L, Gonzalez-Bono E. Salivary cortisol and immunoglobulin A: responses to stress as predictors of health complaints reported by caregivers of offspring with autistic spectrum disorder. Hormones and behavior. 2012;62:464–474. 10.1016/j.yhbeh.2012.08.003 22981424

[8] Hasegawa C, Kikuchi M, Yoshimura Y, Hiraishi H, Munesue T, Takesaki N, et al Changes in autistic trait indicators in parents and their children with ASD: A preliminary longitudinal study. Psychiatry Res. 2015;228:956–957. 10.1016/j.psychres.2015.05.048 26099658

[9] Da Paz NS, Siegel B, Coccia MA, Epel ES. Acceptance or Despair? Maternal Adjustment to Having a Child Diagnosed with Autism. J Autism Dev Disord. 2018. 10.1007/s10803-017-3450-4 PMC6347022 29307036

[10] Pisula E, Porebowicz-Dorsmann A. Family functioning, parenting stress and quality of life in mothers and fathers of Polish children with high functioning autism or Asperger syndrome. PLoS One. 2017;12:e0186536 10.1371/journal.pone.0186536 29036188 PMC5643111

[11] Lloyd TJ, Hastings R. Hope as a psychological resilience factor in mothers and fathers of children with intellectual disabilities. J Intellect Disabil Res. 2009;53:957–968. 10.1111/j.1365-2788.2009.01206.x 19744261

[12] Sheeran T, Marvin RS, Pianta RC. Mothers' resolution of their child's diagnosis and self-reported measures of parenting stress, marital relations, and social support. J Pediatr Psychol. 1997;22:197–212. 9114643 10.1093/jpepsy/22.2.197

[13] Lord B, Ungerer J, Wastell C. Implications of resolving the diagnosis of PKU for parents and children. J Pediatr Psychol. 2008;33:855–866. 10.1093/jpepsy/jsn020 18339641 PMC2493509

[14] Tachibana Y, Miyazaki C, Ota E, Mori R, Hwang Y, Kobayashi E, et al A systematic review and meta-analysis of comprehensive interventions for pre-school children with autism spectrum disorder (ASD). PLoS One. 2017;12:e0186502 10.1371/journal.pone.0186502 29211740 PMC5718481

[15] Smith T, Groen AD, Wynn JW. Randomized trial of intensive early intervention for children with pervasive developmental disorder. Am J Ment Retard. 2000;105:269–285. 10.1352/0895-8017(2000)105<0269:RTOIEI>2.0.CO;2 10934569

[16] Dawson G, Rogers S, Munson J, Smith M, Winter J, Greenson J, et al Randomized, controlled trial of an intervention for toddlers with autism: the Early Start Denver Model. Pediatrics. 2010;125:e17–23. 10.1542/peds.2009-0958 19948568 PMC4951085

[17] Estes A, Munson J, Rogers SJ, Greenson J, Winter J, Dawson G. Long-Term Outcomes of Early Intervention in 6-Year-Old Children With Autism Spectrum Disorder. J Am Acad Child Adolesc Psychiatry. 2015;54:580–587. 10.1016/j.jaac.2015.04.005 26088663 PMC4475272

[18] Schertz HH, Odom SL, Baggett KM, Sideris JH. Mediating Parent Learning to Promote Social Communication for Toddlers with Autism: Effects from a Randomized Controlled Trial. J Autism Dev Disord. 2018;48:853–867. 10.1007/s10803-017-3386-8 29168087

[19] Robins DL, Fein D, Barton ML, Green JA. The Modified Checklist for Autism in Toddlers: an initial study investigating the early detection of autism and pervasive developmental disorders. J Autism Dev Disord. 2001;31:131–144. 11450812 10.1023/a:1010738829569

[20] Allen CW, Silove N, Williams K, Hutchins P. Validity of the social communication questionnaire in assessing risk of autism in preschool children with developmental problems. J Autism Dev Disord. 2007;37:1272–1278. 10.1007/s10803-006-0279-7 17080270

[21] Wainer AL, Hepburn S, McMahon Griffith E. Remembering parents in parent-mediated early intervention: An approach to examining impact on parents and families. Autism. 2017;21:5–17. 10.1177/1362361315622411 26951325

[22] Lord C, Rutter M, DiLavore P, Risi S. Autism Diagnostic Observation Schedule. Los Angeles, CA: Western Psychological Services; 1999.

[23] Wing L, Leekam SR, Libby SJ, Gould J, Larcombe M. The Diagnostic Interview for Social and Communication Disorders: background, inter-rater reliability and clinical use. J Child Psychol Psychiatry. 2002;43:307–325. 11944874 10.1111/1469-7610.00023

[24] American Psychiatric Association, editor. Diagnostic and Statistical Manual of Mental Disorders (DSM-IV). Washington D.C.1994.

[25] Kaufman A, Kaufman N. Kaufman Assessment Battery for Children: Administration and Scoring Manual Circle Pines. 1983;MN: American Guidance Service.

[26] Spielberger CD, Gorsuch RL, Lushene R, Vagg PR, Jacobs GA. Manual for the State-Trait Anxiety Inventory. Palo Alto, CA: Consulting Psychologists Press 1970.

[27] Zung WW. A Self-Rating Depression Scale. Arch Gen Psychiatry. 1965;12:63–70. 14221692 10.1001/archpsyc.1965.01720310065008

[28] The World Health Organization Quality of Life Assessment (WHOQOL): development and general psychometric properties. Soc Sci Med. 1998;46:1569–1585. 9672396 10.1016/s0277-9536(98)00009-4

[29] Matsuoka K, Uno M, Kasai K, Koyama K, Kim Y. Estimation of premorbid IQ in individuals with Alzheimer's disease using Japanese ideographic script (Kanji) compound words: Japanese version of National Adult Reading Test. Psychiatry Clin Neurosci. 2006;60:332–339. 10.1111/j.1440-1819.2006.01510.x 16732750

[30] Deonandan R, Campbell K, Ostbye T, Tummon I, Robertson J. A comparison of methods for measuring socio-economic status by occupation or postal area. Chronic Dis Can. 2000;21:114–118. 11082347

[31] Kamio Y, Inada N, Moriwaki A, Kuroda M, Koyama T, Tsujii H, et al Quantitative autistic traits ascertained in a national survey of 22 529 Japanese schoolchildren. Acta Psychiatr Scand. 2013;128:45–53. 10.1111/acps.12034 23171198 PMC3604131

[32] Constantino JN. The Social Responsiveness Scale: Los Angeles, Calif Western Psychological Services; 2002.

[33] Tani I, Okada R, Ohnishi M, Nakajima S, Tsujii M. Japanese version of home form of the ADHD-RS: an evaluation of its reliability and validity. Res Dev Disabil. 2010;31:1426–1433. 10.1016/j.ridd.2010.06.016 20638822

[34] Ueno K, Nagoshi S, Konuki S. Picture Vocabulary Test-Revised. Hiroshima, Japan: Success/Bell Co Ltd; 2008.

[35] Moriwaki A, Kamio Y. Normative data and psychometric properties of the strengths and difficulties questionnaire among Japanese school-aged children. Child Adolesc Psychiatry Ment Health. 2014;8:1 10.1186/1753-2000-8-1 24444351 PMC3903008

[36] Alhazmi A, Petersen R, Donald KA. Quality of life among parents of South African children with autism spectrum disorder. Acta Neuropsychiatr. 2018:1–6. 10.1017/neu.2018.5 29565002

[37] Niinomi K, Asano M, Kadoma A, Yoshida K, Ohashi Y, Furuzawa A, et al Developing the "Skippu-Mama" program for mothers of children with autism spectrum disorder. Nurs Health Sci. 2016;18:283–291. 10.1111/nhs.12264 26940071

[38] Lindsey RA, Barry TD. Protective Factors Against Distress for Caregivers of a Child with Autism Spectrum Disorder. J Autism Dev Disord. 2018;48:1092–1107. 10.1007/s10803-017-3372-1 29313179

[39] Kim YS, Leventhal BL, Koh YJ, Fombonne E, Laska E, Lim EC, et al Prevalence of autism spectrum disorders in a total population sample. Am J Psychiatry. 2011;168:904–912. 10.1176/appi.ajp.2011.10101532 21558103

[40] Ekas NV, Lickenbrock DM, Whitman TL. Optimism, social support, and well-being in mothers of children with autism spectrum disorder. J Autism Dev Disord. 2010;40:1274–1284. 10.1007/s10803-010-0986-y 20195734

[41] Benson PR. Network characteristics, perceived social support, and psychological adjustment in mothers of children with autism spectrum disorder. J Autism Dev Disord. 2012;42:2597–2610. 10.1007/s10803-012-1517-9 22484793

[42] Derguy C, M'Bailara K, Michel G, Roux S, Bouvard M. The Need for an Ecological Approach to Parental Stress in Autism Spectrum Disorders: The Combined Role of Individual and Environmental Factors. J Autism Dev Disord. 2016;46:1895–1905. 10.1007/s10803-016-2719-3 26858031

[43] Da Paz NS, Wallander JL. Interventions that target improvements in mental health for parents of children with autism spectrum disorders: A narrative review. Clin Psychol Rev. 2017;51:1–14. 10.1016/j.cpr.2016.10.006 27816800

[44] Derguy C, Bouvard M, Michel G, M’Bailara K. The gap between parents’ knowledge and causal beliefs about etiology of autism: A key variable to understand parents' anxiety. European Psychiatry. 2014;29:598–599.

