# Supplementary material for: Changes in maternal feelings for children with autism spectrum disorder after childbirth: The impact of knowledge about the disorder
Source: PLoS One. 2018 Aug 2;13(8):e0201862. doi: 10.1371/journal.pone.0201862 (PMC6072130; doi:10.1371/journal.pone.0201862)
Supplement: S1 Table — (DOCX) [file pone.0201862.s002.docx]

S1 Table 1.

|  | TD | ASD | *t* | *p* |
| --- | --- | --- | --- | --- |
| ***Mothers*** |  |  |  |  |
| Number of subjects | 32 | 28 |  |  |
| Years of age | 38.1 (31-45) | 37.9 (31-50) | 0.2 | n.s. |
| JART (Estimated IQ) | 100.6 (7.8) | 103.5 (8.6) | -1.4 | n.s. |
| SDS | 35.9 (5.2) | 41.0 (9.0) | -2.6 | .001 |
| STAI trait | 39.8 (8.7) | 45.4 (11.9) | -2.1 | .041 |
| QOL (Average) | 3.7 (0.5) | 3.4 (0.6) | 2.0 | n.s |
| QOL subscore I (Physical) | 3.9 (0.6) | 3.5 (0.7) | 2.0 | .048 |
| QOL subscore II (Psychological) | 3.5 (0.5) | 3.3 (0.8) | 1.5 | n.s. |
| QOL subscore III (Social) | 3.8 (0.5) | 3.4 (0.7) | 2.5 | .014 |
| QOL subscore IV (Environment) | 3.6 (0.6) | 3.4 (0.6) | 1.1 | n.s. |
| QOL subscore V (General) | 3.6 (0.7) | 3.2 (0.8) | 2.3 | .024 |
| SES | 42.2 (8.4) | 42.7 (6.2) | -0.3 | n.s. |
| History of psychiatric treatment  (yes/no) | 3/29 | 11/17 | 7.5* | 0.006 |
| ***Children*** |  |  |  |  |
| Gender (male/female) | 19/13 | 23/5 |  |  |
| Chronological age (months) | 70.2 (61-79) | 79.2 (63-111) | -3.1 | .004 |
| Birth weight (g) | 3038.2 (312.9) | 3097.9 (246.4) | -0.8 | n.s. |
| SRS total *T*-score | 47.6 (7.2) | 69.4 (10.6) | -9.4 | P < .001 |
| ADHD-RS | 5.1 (5.0) | 20.8 (10.5)** | -6.8 | P < .001 |
| SDQ | 8.8 (4.5) | 18.4 (5.6)** | -7.1 | P < .001 |
| K-ABC Mental Processing Scale | 105.7 (13.7) | 97.0 (15.0) | 2.4 | .022 |
| PVT-R | 10.5 (3.1) | 9.5 (3.7) | 1.2 | n.s. |

The values represent the mean (range or standard deviation) of each variable. n.s., not significant. *, *X*^2^ value. **, n=24.
